# Comparative studies of mitochondrial proteomics reveal an intimate protein network of male sterility in wheat (*Triticum aestivum* L.)

**DOI:** 10.1093/jxb/erv322

**Published:** 2015-07-01

**Authors:** Shuping Wang, Gaisheng Zhang, Yingxin Zhang, Qilu Song, Zheng Chen, Junsheng Wang, Jialin Guo, Na Niu, Junwei Wang, Shoucai Ma

**Affiliations:** ^1^College of Agronomy, Northwest A&F University, National Yangling Agricultural Biotechnology & Breeding Center, Yangling Branch of State Wheat Improvement Centre, Wheat Breeding Engineering Research Center, Ministry of Education, Key Laboratory of Crop Heterosis of Shaanxi Province, Yangling, Shaanxi 712100, P. R. China; ^2^Institute of Genetics and Developmental Biology, Chinese Academy of Sciences, Beijing 100101, P. R. China

**Keywords:** Male sterility, mitochondrion proteomics, oxidative stress, protein network, tapetal programmed cell death, wheat.

## Abstract

Plant male sterility has often been associated with mitochondrial dysfunction; however, the mechanism in wheat (*Triticum aestivum* L.) has not been elucidated. This study set out to probe the mechanism of physiological male sterility (PHYMS) induced by the chemical hybridizing agent (CHA)-SQ-1, and cytoplasmic male sterility (CMS) of wheat at the proteomic level. A total of 71 differentially expressed mitochondrial proteins were found to be involved in pollen abortion and further identified by MALDI-TOF/TOF MS (matrix-assisted laser desorption/ionization-time of fight/time of flight mass spectrometry). These proteins were implicated in different cellular responses and metabolic processes, with obvious functional tendencies toward the tricarboxylic acid cycle, the mitochondrial electron transport chain, protein synthesis and degradation, oxidation stress, the cell division cycle, and epigenetics. Interactions between identified proteins were demonstrated by bioinformatics analysis, enabling a more complete insight into biological pathways involved in anther abortion and pollen defects. Accordingly, a mitochondria-mediated male sterility protein network in wheat is proposed; this network was further confirmed by physiological data, RT-PCR (real-time PCR), and TUNEL (terminal deoxynucleotidyl transferase-mediated dUTP nick end labelling) assay. The results provide intriguing insights into the metabolic pathway of anther abortion induced by CHA-SQ-1 and also give useful clues to identify the crucial proteins of PHYMS and CMS in wheat.

## Highlight

A mitochondrial protein–protein interaction network in wheat explains why microspores suffered from severe oxidative stress during pollen development, triggered premature tapetal PCD, and consequently resulted in pollen abortion.

## Introduction

Plant male sterility has been observed in numerous species (Schnable and Wise, 1998; Aalto *et al.*, 2013; Bachtrog *et al.*, 2014) since it was first recorded by Kölreuter in 1763. It is characterized by the absence or non-function of plant pollen grains, or the inability of plants to produce or release functional pollen grains. Male-sterile mutants (including physiological male-sterility; PHYMS) are of tremendous value as genetic tools in male development research and the use of heterosis (Eckardt, 2006; Ba *et al.*, 2014a). Despite it being suspected that it is closely related to mitochondrial function (Linke and Borner, 2005; J.W. Wang *et al.*, 2013; Heng *et al.*, 2014; Touzet and Meyer, 2014), the genetic and molecular mechanisms of male sterility in wheat remain to be further elucidated.

Mitochondria play a pivotal role in energy metabolism and the maintenance of cellular homeostasis, and their dysfunction is associated with male sterility in plants (Eckardt, 2006; Kumar *et al.*, 2012; Chen and Liu, 2014; Horn *et al.*, 2014; Hu *et al.*, 2014; Touzet and Meyer, 2014). More recently, with the mitochondrial genome sequence completely elucidated in some higher plant species, there has been an increased application of genomic approaches to study anther development and pollen reproduction, for example in the monocotyledonous plants *Oryza sativa* (rice) (Notsu *et al.*, 2002), *Zea mays* (maize) (Clifton *et al.*, 2004), and *Triticum* species (wheat) (Ogihara *et al.*, 2005), and dicotyledonous plants such as *Arabidopsis thaliana* (Unseld *et al.*, 1997), *Brassica napus* (Handa, 2003), and *Nicotiana tabacum* (tobacco) (Sugiyama *et al.*, 2005). Many studies suggest that the mitochondrial genome is the carrier of fertility-related genes (Hanson and Bentolila, 2004; Chen *et al.*, 2010; Luo *et al.*, 2013; Tang *et al.*, 2014). Some of these genes and/or open reading frames (ORFs) encode pollen abortion-related proteins, such as ORF256 (7kDa) (Song and Hedgcoth, 1994), ORF13 (13kDa) (Klein *et al.*, 2005), ORF138 (19kDa) (Yasumoto *et al.*, 2009), ORF288 (32kDa) (Jing *et al.*, 2012), ORF79 (8.9kDa) (Wang *et al.*, 2006; Luan *et al.*, 2013), PCF (25kDa) (Bentolila *et al.*, 2002), and WA352 protein (Luo *et al.*, 2013; Tang *et al.*, 2014). However, how these proteins mediate metabolic-induced male sterility still needs to be elucidated, and the protein–protein interaction network remains largely unexplored, especially in wheat.

In the current study, physiological male-sterile Xinong 1376 (PHYMS-XN1376) plants (Zhang *et al.*, 2013; Ba *et al.*, 2014a, b; Liu *et al.*, 2014), cytoplasmic male sterile Xinong 1376 (CMS-XN1376) plants, and their maintainer line (MF-XN1376) (Chen *et al.*, 2009, 2010) were used. The isolation of floret mitochondria from wheat using Percoll density gradient methods is described. This is followed by the application of two-dimensional electrophoresis (2-DE) in combination with tandem mass spectrometry (MS/MS) analysis to compare the different expression patterns of floret mitochondrial proteins at the early uninucleate stage and the trinucleate stage. Seventy-one mitochondrial differentially expressed proteins (DEPs) were found to be involved in pollen abortion. Identification of these proteins combined with their changes in abundance as well as the impact of pollen development establishes a close linkage between mitochondria and male sterility. In the present study, a global view of the ubiquitous cellular changes associated with pollen abortion is revealed, and the existence of an intimate protein network elicited by mitochondrial mutation in male-sterile wheat is suggested. This network was further confirmed with other molecular biology techniques. The results provide insights into the molecular events of male sterility.

## Materials and methods

### Plant material and treatment

PHYMS-XN1376 plants were treated with the new type of chemical hybridizing agent (CHA)-SQ-1; it has broad-spectrum properties and can lead to complete male sterility, as has been shown through pharmacodynamic experimental field tests. It also has no side effects on agronomic traits. Currently, SQ-1 has been used to produce hybrid seeds on a large scale, as has been previously described (Ba *et al.*, 2014a; Song *et al.*, 2014). The PHYMS-XN1376, CMS-XN1376, and MF-XN1376 plants with the same nuclear background (*T. aestivum* nucleus) were grown conventionally in wheat fields at the experimental station of the Northwest Agriculture and Forestry University, Yangling, China (108°E, 34°15’N). The pollen developmental stages have been described previously (Song *et al.*, 2014). Florets from which glumes and awns were removed (at the early uninucleate stage and the trinucleate stage, respectively) were harvested for isolation of mitochondria. Anthers at sequential stages (early uninucleate stage, binucleate stage, and trinucleate stage, respectively) were also collected.

### Phenotypic characterization

Plant materials were photographed with a Nikon E995 digital camera (Nikon, Japan) mounted on a Motic K400 dissecting microscope (Preiser Scientific, Louisville, KY, USA). Fresh microspores were stained with 1% acetocarmine and 2% iodine–potassium iodide (2% I_2_–KI). For scanning electron microscopy, anthers were collected and processed essentially as described by Xu *et al.* (2014) and Zhang *et al.* (2010), and observed with a JSM-6360LV scanning electron microscope (JEOL).

### Preparation of wheat mitochondria

Mitochondria were isolated from florets according to Chen *et al.* (2010) with minor modifications. Briefly, ~70g (fresh weight) of florets were homogenized with 700ml of ice-cold homogenization solution [0.3M mannitol, 50mM Na_4_P_2_O_7_, 2mM EGTA, 0.5% (w/v) polyvinylpyrrolidone (PVP)-40, 0.5% (w/v) bovine seum albumin (BSA), 20mM ascorbate, pH 8.0] using a pre-cooled Waring blender. After filtering through four layers of Miracloth (Calbiochem, San Diego, CA, USA), the filtrates were centrifuged at 3000 *g* for 15min, and then the supernatant was centrifuged at 18 000 *g* for 20min. The resultant organelle pellet was resuspended in 300ml of wash buffer [0.3M mannitol, 10mM TES-NaOH (pH 7.5), and 0.1% (w/v) BSA] with the aid of a clean soft-bristle paint brush, and the two centrifugation steps were repeated to produce a washed organelle pellet. The final organelle pellet was resuspended in 10ml of wash buffer and loaded onto a three-step Percoll gradient composed of, from bottom to top, 40% (v/v) Percoll, 24% (v/v) Percoll, and 20% (v/v) Percoll, each Percoll solution containing 0.3M mannitol, 0.1% (w/v) BSA, and 10mM TES-NaOH (pH 7.5), and centrifuged at 40 000 *g* for 50min. The light yellow-brown bands of mitochondria at the 40% and 24% interfaces were collected with an injection needle and washed through 5× dilutions in wash medium, and then centrifuged at 20 000 *g* for 10min. This was further purified by centrifugation at 40 000 *g* for 30min in a one-step Percoll gradient [28% Percoll in buffer containing 0.3M sucrose, 10mM TES-KOH (pH 7.5), and 0.1% (w/v) BSA]. The upper mitochondrial fraction was collected, washed twice by 1× dilutions in wash buffer (minus 0.1% BSA), and centrifuged at 20 000 *g* for 10min. The mitochondria-enriched pellet was resuspended in ~500 μl of wash buffer (minus 0.1% BSA), and used for the isolation of mitochondrial proteins. Mitochondrial activity was stained with 0.01M Janus green B as described previously (Chen *et al.*, 2010), the integrity was assessed using a negative stain for transmission electron microscopy (TEM; Hitachi HT7700, Tokyo, Japan).

### Two-dimensional gel electrophoresis

2-DE was performed following the method of Ye *et al.* (2013). Mitochondria were solubilized in 500 μl of lysis solution containing 7M urea, 2M thiourea, 4% (w/v) CHAPS, 65mM dithiothreitol (DTT), 0.5% (v/v) Bio-Lyte (pH 4–7), and 0.001% (w/v) bromphenol blue. After determining the protein concentration using the Bradford method as described by Zuo and Lundahl (2000), ~160 μg of mitochondrial protein was separated by loading the sample on a 17cm pH 4–7 linear immobilized pH gradient (IPG) strip (Bio-Rad) and actively rehydrated at 50V for 12h at 20 °C. Subsequently, focusing was performed using the IPGphor Isoelectric Focusing System apparatus under the following conditions: constant power (50 μA per IPG strip) at 250V for 1h, 500V for 1h, 1000V for 1h, 8000V for 4h, and 8000V for a total of 80 000 V-h (17cm, pH 4–7). The second electrophoretic dimension took place using 11% SDS–PAGE. Protein spots were visualized by silver staining.

### Gel imaging and data analysis

Protein spots were analysed with PDQuest Software, version 8.0.1 (Bio-Rad) according to the manufacturer’s instructions. Briefly, the images were initially processed through transformation, filtering, automated spot detection, normalization, and matching. All protein spots detected in-gel were matched to the corresponding spots of the master gel (Euns-MF) and each spot density was normalized against the whole gel densities. The volume of the spot corresponded to the amount of protein expressed. Normalized spot volumes were used to compare different samples statistically and to determine fold change values. Only the spots altered >1.3-fold (*P*≤0.05) were considered as DEPs.

### In-gel digestion and MALDI-TOF/MALDI-TOF-TOF MS analysis

DEPs detected in silver-stained gels were selected and excised manually for protein identification. In-gel digestion of protein spots was essentially performed according to the method of Ye *et al.* (2013). Matrix-assisted laser desorption/ionization (MALDI) samples were prepared according to a thin layer method as described by Gobom *et al.* (2001). Mass spectra were collected with a 5800 MALDI time of flight (TOF)/TOF™ analyser and analysed using TOF/TOF™ Series Explorer^TM^ Software V4.1.0 (AB Sciex, Foster City, CA, USA). Mass spectrometry (MS) spectra were recorded in a mass range from 700Da to 4000Da with a focus mass of 1700Da. For one main MS spectrum, 15 subspectra with 200 shots per subspectrum were accumulated, and for the MS/MS spectrum up to 25 subspectra with 250 shots per subspectrum were accumulated. Peptide mass fingerprinting (PMF) and MS/MS data were used to derive protein identity using the MASCOT search engine (Matrix Science) applied to the NCBInr or Swiss-Prot databases. The main parameters were set as follows: S/N ≥3.0; fixed modification, carbamidomethyl (Cys); variable modification, Gln→pyro-Glu (N-term Q), and oxidation (Met); maximum number of missing cleavages, 1; MS tolerance, ±100 ppm.; and MS/MS tolerance, ±0.3Da. Other search parameters are stated in the protein identification tables (Supplementary Table S1 available at *JXB* online).

### Bioinformatic analysis of identified proteins

The function of the identified proteins was elucidated by using the gene index accompanied Uniprot accession number as input for the Uniprot database (http://www.uniprot.org). Hierarchical clustering was performed with the log-transformed data using Genesis 1.7.6 software. To better understand functions and interactions of identified proteins, a protein–protein interaction network was predicted. All identified proteins were blasted against the *A. thaliana* TAIR10 (http://www.arabidopsis.org/) and rice protein databases with the intention of obtaining annotated protein entries for protein–protein interaction tools (Jacoby *et al.*, 2010). Interactions of the identified proteins were constructed with the online analysis tool STRING 9.1 (Szklarczyk *et al.*, 2015). Cellular component and biological processes were predicted by Cytoscape plugin BiNGO 3.02 (Maere *et al.*, 2005). The search parameters, TAIR and rice homologous proteins are listed in Supplementary Table S2 at *JXB* online.

### Respiratory activity measurements

The activity of the cytochrome oxidase pathway (COP; *V*_cyt_) in anther was calculated by multiplying the total respiration rate (*V*_t_) and measured using the method of Vanlerberghe and McIntosh (1996).

### Measurement of physiological indexes

O_2_^–^ and H_2_O_2_ content, and superoxide dismutase (SOD), catalase (CAT), and guaiacol peroxidase (POD) activity were established using the method of Gajewska and Sklodowska (2007). Lipid peroxidation was determined by estimating the malondialdehyde (MDA) content according to Hameed *et al.* (2012, 2014).

### RT-PCR analysis

All primers are listed in Supplementary Table S3 at *JXB* online. Total RNA was isolated from anthers at different stages with the Trizol Reagent Kit (Invitrogen, Carlsbad, CA, USA) and subjected to first-strand cDNA synthesis using a PrimeScript™ RT reagent Kit (Takara Bio, Tokyo, Japan), according to the manufacturer’s protocol. To obtain reliable results, the Ct value was obtained from CFX96™ Real-Time PCR Detection System (Bio-Rad) using SYBR Premix EX Taq (Takara Bio). The geNorm and BestKeeper method algorithms (RefFinder, http://www.leonxie.com/referencegene.php?type=reference) were used to select the most suitable reference gene from the seven candidates. Both programs demonstrated that *Actin* was the best reference gene for quantifying gene expression (Supplementary Fig. S1). The RT-PCR was performed with TaKaRa Ex Taq DNA polymerase (Takara Bio) for 37 cycles of denaturation for 40 s at 94 °C, annealing for 40 s at 50–60 °C, and extension for 30 s at 72 °C, followed by a final extension for 10min at 72 °C. The PCR products were identified by 2% (w/v) agarose gels and visualized by ethidium bromide staining.

### TUNEL assay

Anther sections (6 μm) were washed in phosphate-buffered saline (PBS) for 5min and fixed in 4% (w/v) paraformaldehyde in PBS for 15min, and then incubated in 20 μg ml^–1^ proteinase K (Promega) in PBS for 10min. Terminal deoxynucleotidyl transferase-mediated dUTP nick end labelling (TUNEL) was performed in a humid chamber for 60min in the dark at 37 °C using a Dead End Fluorometric TUNEL Kit (Promega, Madison, WI, USA) following the manufacturer’s instructions. All reactions were counterstained with 1 μg ml^–1^ propidium iodide (PI; Promega) in PBS for 15min to label all nuclei. Samples were analysed under a fluorescence confocal scanner microscope (A1R; Nikon, Tokyo, Japan). The green fluorescence of fluorescein (TUNEL signal) and red fluorescence of PI were analysed at 488nm (excitation) and 520nm (detection), and 488nm (excitation) and 620nm (detection), respectively.

## Results

### Validation of morphological defects in male-sterile mutants

**Fig. 1.**
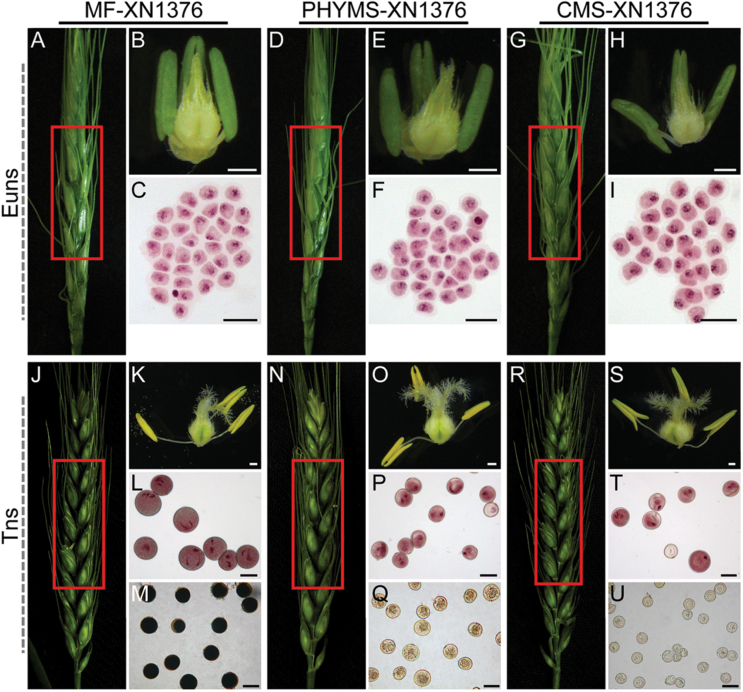
Comparison of morphological features of PHYMS-XN1376, CMS-XN1376, and their maintainer line MF-XN1376. Comparison of spikes (A, D, G, J, N, R), and anthers and pistils (B, E, H, K, O, S) of CMS-XN1376, PHYMS-XN1376, and MF-XN1376 plants. The 1% acetocarmine (C, F, I, L, P, T) and 2% I_2_–KI (M, Q, U) staining of pollen grains. Euns, the early uninucleate stage; Tns, the trinucleate stage. Scale bars=1mm (B, E, H, K, O, S), 100 μm (C, F, I, L, M, P, Q, T, U).

The morphological features of MF-XN1376, PHYMS- XN1376, and CMS-XN1376 plants were compared. All of these materials have the same nuclear background (*T. aestivum* nucleus). Compared with MF-XN1376, PHYMS-XN1376 and CMS-XN1376 showed normal spike and floral development but failed to produce any viable pollen at different stages of pollen development (Fig. 1A, D, G, J, N, R); however, the pistils of PHYMS-XN1376 and CMS-XN1376 were normal and able to produce normal seeds when they were backcrossed with fertile pollen (Fig. 1B, E, H, K, O, S). At the early uninucleate stage, there were no visible differences in pollen among the three samples after staining with 1% acetocarmine (Fig. 1C, F, I); however, the anthers of CMS-XN1376 were detectably different from the others (Fig. 1B, E, H), and there were no visible differences between MF-XN1376 and PHYMS-XN1376 (Fig. 1B, E). At the trinucleate stage, compared with MF-XN1376 anthers, the sterile anthers from both PHYMS-XN1376 and CMS-XN1376 plants were small and without mature pollen grains (Fig. 1K, O, S; Supplementary Fig. S2 at *JXB* online). Futhermore, fertile pollen grains of MF-XN1376 could be clearly distinguished from the sterile pollen of PHYMS-XN1376 and CMS-XN1376 by staining with 1% acetocarmine (Fig. 1L, P, T) and 2% I_2_–KI (Fig. 1M, Q, U). These results indicated that PHYMS-XN1376 and CMS-XN1376 plants were 100% pollen sterile.

### Isolation of high-purity mitochondria from wheat florets

The isolation of pure organellar fractions from crude source material is the critical phase of any subproteome analysis. It was previously established that very pure mitochondria can be obtained from wheat (Chen *et al.*, 2010). This established procedure was employed for the isolation of mitochondria with a recognized low contamination level. The function and integrity of the purified mitochondria were also assayed to ensure that proteins were not being lost through rupture during isolation and that key functions were maintained. Photomicrographs of mitochondria-enriched fractions generated with Percoll gradients are presented in Supplementary Fig. S3 at *JXB* online. The activity of the purified mitochondria was visualized using Janus green B staining under a light microscope (Supplementary Fig. S3A); they retained high activity after the 28% Percoll self-forming density centrifugation. The structural integrity of the purified mitochondria was confirmed by electron microscopy where most appeared morphologically intact (Supplementary Fig. S3B, C).

### Establishment and analysis of mitochondrial proteome maps for male-sterile mutants and their maintainer line

Three independent samples of mitochondrial proteins isolated from wheat were separated by 2-DE (Supplementary Fig. S4 at *JXB* online). From a spot-to-spot comparison and statistical analysis, a total of 109, 105, and 128 reproducible protein spots were detected at the early uninucleate stage from MF-XN1376, PHYMS-XN1376, and CMS-XN1376 plants, respectively (Table 1; Supplementary Fig. S4A–C). The 2-DE gels also detected 129, 180, and 159 reproducible protein spots at the trinucleate stage in MF-XN1376, PHYMS-XN1376, and CMS-XN1376 plants, respectively (Table 1; Supplementary Fig. S4D–F). Table 1 presents the comparison of mitochondrial proteomics using different materials and an analysis of how these spots overlapped. Not surprisingly, there were large overlaps between these spots. In the same stages of these three materials, 96 spots overlapped at the early uninucleate stage in all three materials and 127 spots overlapped at the trinucleate stage. Additionally, in the same materials of these two stages, 101 spots in MF-XN1376, 94 spots in PHYMS-XN1376, and 119 spots in CMS-XN1376 overlapped between the two stages. Overall, 92 overlapping spots were seen in all of the 2-DE gels (Table 1; Supplementary Fig. S4).

**Table 1.** Comparison of mitochondrial proteomics at the early uninucleate and trinucleate stages of the *MF-XN1376, PHYMS-XN1376*, and *CMS-XN1376* varieties of wheat

Pollen developmental stages	MF-XN1376	PHYMS-XN1376	CMS-XN1376	Overlapped
Euns	109	105	128	96
Tns	129	180	159	127
Overlapped	101	94	119	92

Overlapped; proteins showing the same expression patterns in both the early uninucleate stage (Euns) and trinucleate stage (Tns) of PHYMS-XN1376, CMS-XN1376, and MF-XN1376, respectively, or among MF-XN1376, PHYMS-XN1376, and CMS-XN1376 at the early uninucleate stage or the trinucleate stage.

### Differentially expressed proteins among male-sterile mutants and their maintainer line

**Fig. 2.**
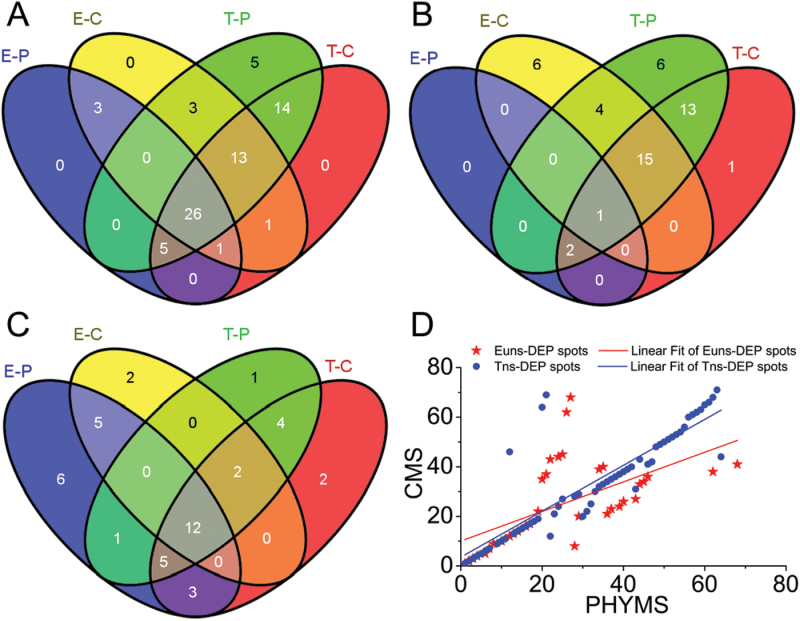
Venn diagram and scatter plot analysis of DEP spots in PHYMS-XN1376, CMS-XN1376, and MF-XN1376. The numbers of DEP spots which are up- or down-regulated are shown in the different segments; more details are presented in Supplementary Table S5 at *JXB* online. (A) All of the DEP spots; (B) the up-regulated protein spots; (C) the down-regulated protein spots. E/T-P, the early uninucleate stage/trinucleate stage of PHYMS-1376; E/T-C, the early uninucleate stage/trinucleate stage of CMS-XN1376. (D) The PHYMS-XN1376/CMS-XN1376 ratio of each DEP at the early uninucleate and trinucleate stages from PHYMS-XN1376 is plotted on the *x*-axis and correspondingly that from CMS-XN1376 is plotted on the *y*-axis.

To determine the proteins whose abundance changed in male-sterile mutants, a threshold limit of 1.3-fold (*P*≤0.05) was set in this study as previously reported (Shen *et al.*, 2009). Statistical analysis of 2-DE gels revealed that 71 protein spots (numbered from 1 to 71) were differentially expressed among MF-XN1376, PHYMS-XN1376, and CMS-XN1376 plants in both the early uninucleate stage and the trinucleate stage (Supplementary Figs S4, S5; Tables S4, S5 at *JXB* online). Of these, at the early uninucleate stage, there were 35 DEPs in PHYMS-XN1376 (three up- and 32 down-regulated) and 47 DEPs in CMS-XN1376 (21 up- and 26 down-regulated) (Fig. 2A–C; Supplementary Table S5). At the trinucleate stage, there were 66 DEPs in PHYMS-XN1376 (41 up- and 25 down-regulated) and 60 DEPs in CMS-XN1376 (32 up- and 28 down-regulated) (Fig. 2A–C; Supplementary Table S5). Additionally, overlapping DEPs were detected in all of the male-sterile plants, from 30 at the early uninucleate stage up to 58 at the trinucleate stage (Fig. 2A; Supplementary Table S5), and had a corresponding Pearson correlation coefficient from 0.33 up to 0.73 with a *P*-value <0.01 (Fig. 2D). Therefore, these observations document the changes in abundance of these DEPs along with the occurrence of pollen abortion. These changes show that there are different ways to induce male sterility under mitochondrial protein regulation in the initial stage of anther abortion, and ultimately this regulation makes these ways converge.

### Identification and functional classification of DEPs

In order to analyse further the physiological and biochemical properties of different proteins and their relationship to the abortive process, a total of 71 DEPs were analysed by MALDI-TOF/TOF MS (Supplementary Tables S1, S6 at *JXB* online). These proteins, associated with pollen development and tapetal activities in other plants, were found to be differentially expressed in PMS-XN1376 and CMS-XN1376 plants, as shown in Supplementary Tables S1 and S6. Among the 71 identified spots, 68 (~96%) have been functionally annotated in the current database, whereas three (spots 69–71) were annotated as unknown. To annotate these identities, their sequences were used as queries to search for homologues by BLASTP (NCBI). The corresponding homologues with the highest similarity are listed in Supplementary Table S7. All three proteins shared at least 80% positive identity with their homologues at the amino acid level, suggesting that they may have similar function. However, about one-third of the identified proteins were detected in multiple spots with different pIs or molecular masses (Supplementary Table S1), implying the existence of isoforms and post-translational modification.

**Fig. 3.**
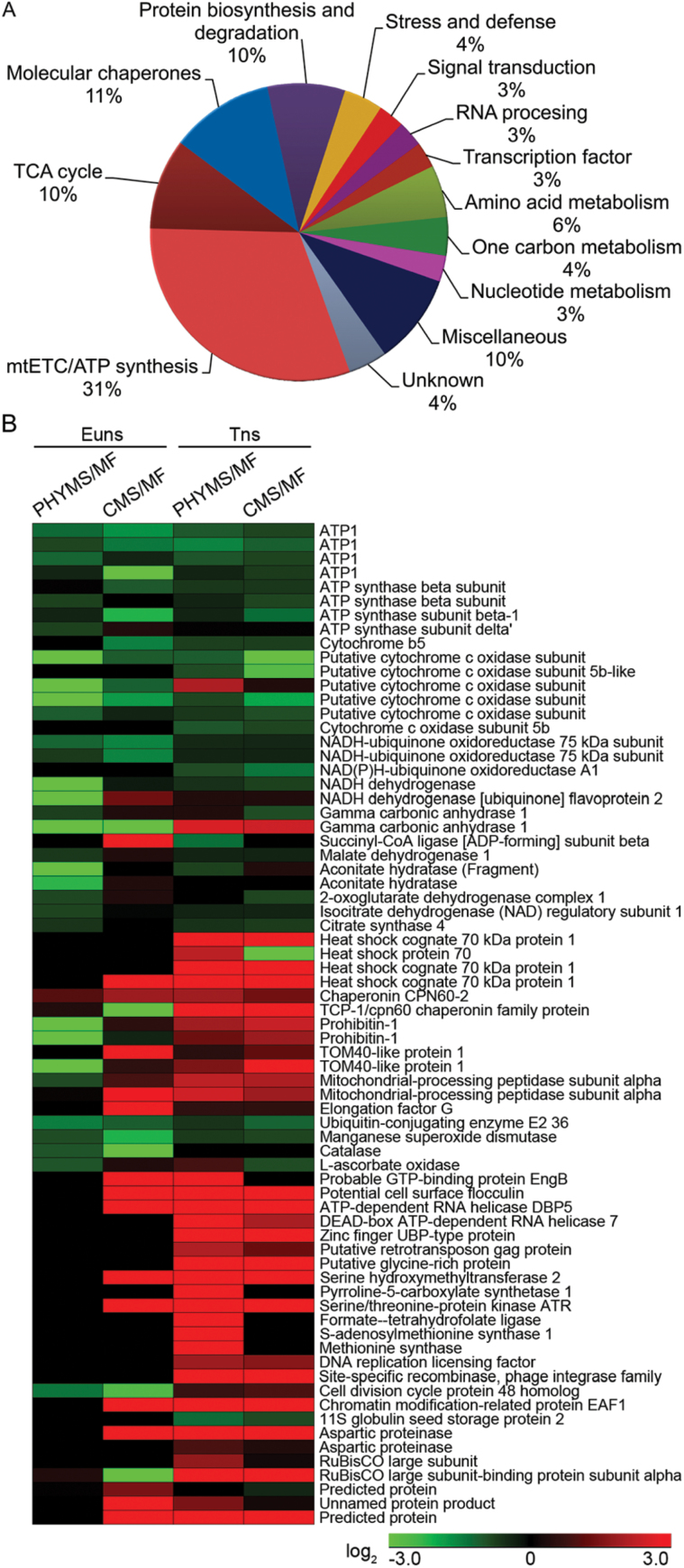
Functional classification (A) and hierarchical clustering (B) of the identified proteins from florets of PHYMS-XN1376, CMS-XN1376, and MF-XN1376 at the early uninucleate and trinucleate stages. The hierarchical cluster analysis was conducted using the Genesis 1.7.6 procedure (Graz University of Technology, Austria, http://genome.tugraz.at/genesisclient/genesisclient_download.shtml) and the log_2_-transformed values of abundance change presented in Supplementary Fig. S5 and Table S5 at *JXB* online. Euns, the early uninucleate stage; Tns, the trinucleate stage; PHYMS, PHYMS-XN1376; CMS, CMS-XN1376; MF, MF-XN1376.

Furthermore, based on the biological process of each gene product according to annotations in these databases coupled with the metabolic and functional features of pollen, all of these identities were classified into 12 functional groups (Fig. 3A; Supplementary Table S1 at *JXB* online), which covered a wide range of pathways and functions, including mitochondrial electron transport (mtETC) and ATP synthesis, the tricarboxylic acid (TCA) cycle, molecular chaperones, protein biosynthesis and degradation, stress and defence, signal transduction, RNA processing, transcription factors, amino acid metabolism, one-carbon metabolism, and nucleotide metabolism. An impressive 62% of these identified proteins were implicated in the first four functional groups, whereas the largest functional group was proteins related to mtETC/ATP synthesis (31%). The other three predominant categories were proteins related to the TCA cycle (10%), molecular chaperones (11%), and protein biosynthesis and degradation (10%). Details of the function of the identified proteins and their abundance profiles in PHYMS-XN1376 and CMS-XN1376 (Fig. 3B) are discussed later.

### Protein–protein interaction analysis of identified proteins

Identified proteins were grouped into functional classes according to the biological processes in which they are involved. STRING and BiNGO, which offer an upgrade of the functional analysis, were used to visualize the protein–protein interaction, cellular component, and biological pathways. The STRING analysis revealed a protein association network (Supplementary Fig. S6 at *JXB* online), in which proteins associated with mtETC, ATP synthesis, the TCA cycle, protein metabolism, the *S*-adenosylmethionine (SAM) cycle, and antioxidant activity were highly clustered, and these clusters are highlighted with ellipses in Supplementary Fig. S6. Abbreviations of the specific protein names in the network are given in Supplementary Table S8. To obtain statistically over- or under-represented categories of cellular components and biological pathways related to male sterility, BiNGO was used to analyse the identified proteins (Supplementary Fig. S7). The results of cellular component analysis indicated that the identified proteins were more highly enriched in mitochondria (*P*=5.99e–24; Supplementary Fig. S7A; Table S9), but a number of non-mitochondrial proteins were identified in this study, which is a common problem in isolating mitochondria from plant tissue and also observed in many other mitochondrial proteomic studies (Taylor *et al.*, 2005; Jacoby *et al.*, 2010; Salvato *et al.*, 2014). A complete list of the enriched Gene Ontology (GO) biological pathways for the proteins is presented in Supplementary Fig. S7B and Table S10. Of these, metabolic process (*P*=5.90e–6) and response to stimulus (*P*=7.28e–11) were significantly over-represented. Moreover, response to chemical stimulus (*P*=2.45e–11) was highly correlated with PHYMS-XN1376. Two other groups could be observed both in metabolic process and response to stimulus, namely response to oxidative stress (*P*=6.41e–7) and oxygen and reactive oxygen species metabolic process (*P*=3.90e–4), implying that male-sterile plants suffered from oxidative stress. The putative implications for the identified proteins in male sterility are discussed below. The network clusters obtained can provide a broader insight into the different roles of the identified proteins. These proteins are included in a wide range of biological pathways that are involved, either directly or indirectly, in male sterility of wheat.

### Oxidative stress and tapetal programmed cell death (PCD) in anther

Mitochondria are not only the sites of oxygen consumption but also one of the sources of cellular reactive oxygen species (ROS) (Salvato *et al.*, 2014). From the above results, the DEPs limited the electron transfer process and this was further confirmed by respiration assays (Supplementary Fig. S8 at JXB online); this might disrupt the balance of oxidative stress, and further trigger PCD in the anther. Therefore, to confirm these possibilities further, the ROS levels and DNA damage were analysed.

**Fig. 4.**
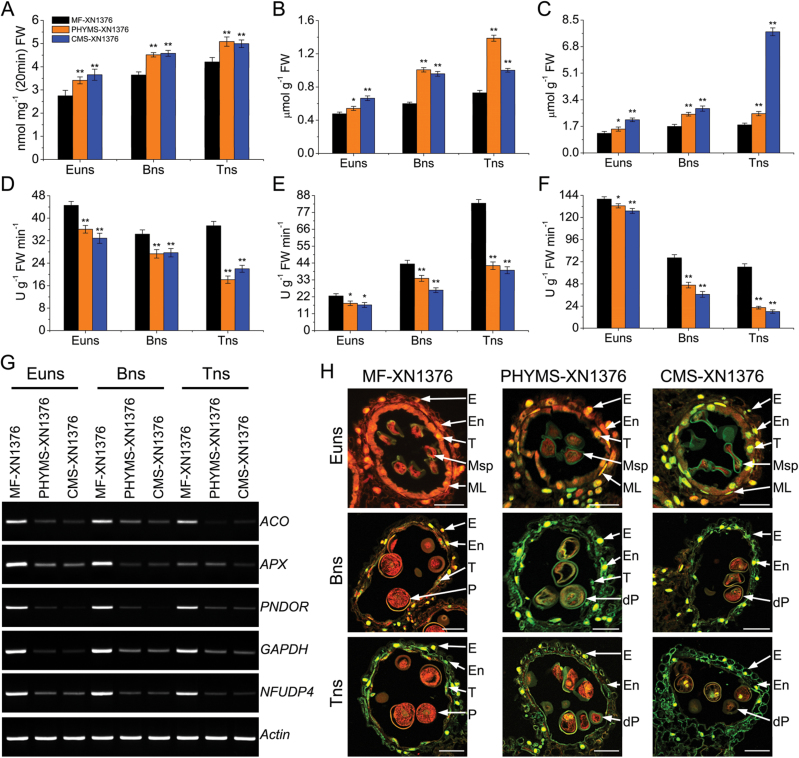
ROS burst induces premature tapetal PCD. (A–F) The O_2_^–^ production rate (A), H_2_O_2_ (B), and MDA (C) contents, and the activities of SOD (D), POD (E), and CAT (F) in developing anthers. Data are means ±SD of three independent experiments. The significance of differences was assessed by Student’s *t*-test (*P<0.05, **P<0.01). Euns, the early uninucleate stage; Bns, the binucleate stage; Tns, the trinucleate stage. (G) Analysis of oxidation stress and energy supply genes by RT-PCR assay. Primers are listed in Supplementary Table S3 at *JXB* online. ACO, aconitase; APX, ascorbate peroxidase; PNDOR, pyridine nucleotide-disulphide oxidoreductase; GAPDH, glyceraldehydes-3-phosphate dehydrogenase; NFUDP4, nifU-like domain protein 4. (H) Detection of premature PCD by TUNEL assay in the anthers at different developmental stages. Red fluorescence represents staining with propidium iodide, and green fluorescence indicates TUNEL-positive nuclei of PCD cells. E, epidermis; En, endothecium; ML, middle layer; T, tapetum; Msp, microspore; P, pollen; dP, degenerated pollen. Scale bar=100 μm.

ROS contents and antioxidant enzyme activities were first measured (Fig. 4A–C). The levels of O_2_^–^ in PHYMS-XN1376 and CMS-XN1376 anthers were significantly higher than in MF-XN1376 from the early uninucleate stage to the trinucleate stage (Fig. 4A); the levels increased rapidly and remained 20% higher in PHYMS-XN1376, and reached a maximum value of 133% in CMS-XN1376. Excess O_2_^–^ was catalysed to form H_2_O_2_. The change of H_2_O_2_ content was similar to that of O_2_^–^; it apparently increased with anther development in PHYMS-XN1376 and reached a maximum value of 190% at the trinucleate stage, and remained 35% higher in CMS-XN1376 (Fig. 4B). ROS scavenging depends on antioxidant enzymes, such as SOD, CAT, and POD. However, in this experiment, the activities of these enzymes were significantly decreased in PHYMS-XN1376 and CMS-XN1376 anthers when excess ROS were generated (Fig. 4D–F), and this further disrupted the oxidative/antioxidative balance. Subsequently, excess ROS degraded polyunsaturated lipids to form MDA, and its level was significantly increased in anthers of PHYMS-XN1376 (maximum value of 147%) and CMS-XN1376 (maximum value of 432%) (Fig. 4C). Moreover, RT-PCR analysis showed that two oxidation stress-related genes [ascorbate peroxidase (APX) and pyridine nucleotide-disulphide oxidoreductase (PNDOR)] were down-regulated in PHYMS-XN1376 and CMS-XN1376 (Fig. 4G). The above results indicate that the production of ROS overwhelmed the antioxidant capacity, which disrupted the balance between ROS production and clearance in male-sterile anthers; therefore oxidative stress occurred. Therefore, these results suggest that the oxidative stress of the anther in PHYMS-XN1376 was the same as that in CMS-XN1376.

As described above, the ROS levels exhibited a time-dependent increase in male-sterile anthers. Excessive ROS *in vivo* may generate intermediate signals involved in PCD (Maxwell *et al.*, 2002; Y. Wang *et al.*, 2013). To investigate the abnormal PCD under oxidative stress in the anthers of PHYMS-XN1376 and CMS-XN1376 TUNEL, assays were then performed on anther sections (Fig. 4H; Supplementary Fig. S9 at *JXB* online). At the early uninucleate stage, the TUNEL fluorescence signal was detected in both PHYMS-XN1376 and CMS-XN1376 tapetal cells, suggesting that PCD occurred in the tapetum and was more strongly expressed in CMS-XN1376 than in PHYMS-XN1376. However, the TUNEL fluorescence signal was still not detected in microspores. Subsequently, from the binucleate stage to the trinucleate stage, TUNEL fluorescence showed stronger signals in the male-sterile tapetum and microspores. Then, the tapetal cells degraded completely and disappeared in PHYMS-XN1376 at the trinucleate stage and in CMS-XN1376 at the binucleate stage. These observations demonstrated that tapetum PCD commenced at the early uninucleate stage and it was degraded prematurely in PHYMS-XN1376 and CMS-XN1376. Furthermore, DNA fragmentation was apparently synchronized with excessive ROS levels.

These results indicated that down-regulation of DEPs inhibited the mtETC. This inhibition was correlated with excess production of ROS and reduced activity of antioxidant enzymes; the microspores suffered from severe oxidative stress during pollen development. Then, the chronic oxidative stress triggered PCD and consequently resulted in the abortion of microspores.

## Discussion

### DEPs preferentially disturbed the mtETC and ATP synthesis

Among the 12 functional groups, the largest of them was composed of proteins involved in mtETC/ATP synthesis, including 22 down-regulated proteins as listed in Supplementary Table S1 at *JXB* online (spots 1–22).

There were 12 proteins participating in mtETC (spots 9–20). In mitochondria, NADH dehydrogenase and NADH-ubiquinone oxidoreductase (also referred to as complex I) are the first and the largest enzyme of the mtETC. NADH initially binds to complex I and transfers high energy electrons to subsequent complexes (complex III. complex IV, and complex V). Remarkably, complex I is one of the main sites of premature electron leakage to oxygen (Moller, 2001) and has a role in triggering apoptosis (Petrussa *et al.*, 2009; Chomova and Racay, 2010). In the present study, in both PHYMS-XN1376 and CMS-XN1376 plants, their down-regulation (spots 9–20; Fig. 3B; Supplementary Fig. S5 at *JXB* online) limited the electron transfer process, promoting ROS generation (Fig. 4A–C). If those ROS were not scavenged, superabundant ROS would then cause oxidative damage to cells, resulting in protein and DNA damage, lipid peroxidation, and even cell death (Foyer and Noctor, 2005). Here an inverse correlation was found between the level of intracellular ROS and the activities of anther SOD (Fig. 4D), CAT (Fig. 4E), and POD (Fig. 4F). Furthermore, applying the TUNEL assay, it was observed that anthers of PHYMS-XN1376 and CMS-XN1376 showed typical PCD characteristics (Fig. 4H; Supplementary Fig. S9). Additionlly, six COX subunits (spots 10–15) were down-regulated in two male sterility samples; this altered cytochrome *c* oxidase functionality or structure, which in turn inhibits electron transport from cytochrome *c* to oxygen. Therefore, the decreased abundance of the 12 proteins limited the mtETC and further affected pollen development.

Furthermore, representative subunits of ATP synthase were identified, including four α-subunits (spots 1–4), three β-subunits (spots 5–7), and one δ-subunit (spot 8). Mitochondrial membrane ATP synthase (F_1_F_0_ ATP synthase or complex V) plays an important role in energy metabolism by converting ADP into ATP in the presence of a transmembrane proton gradient (Sabar *et al.*, 2003). The β-subunit is a catalytic site, which in combination with the α-subunit regulates the activity of ATP synthase. Recent studies of plant mitochondrial complexes indicate that several mitochondrial DNA regions encoding these ATP synthase subunits are associated with male sterility (Sabar *et al.*, 2003; Eckardt, 2006; Ohta *et al.*, 2010). In this study, similar patterns were also observed for these subunits (spots 1–8) that decreased in both PHYMS-XN1376 and CMS-XN1376 mitochondria (Fig. 3B; Supplementary Fig. S5 at *JXB* online). These might cause ATP synthase dysfunction and then impact on mitochondrial energy output and induce mitochondrial membrane potential changes, resulting in abnormal anther development with non-functional pollen.

Therefore, the present data suggest that the down-regulation of the 22 proteins not only slowed down the rate of electron transport in both PHYMS-XN1376 and CMS-XN1376 plants, but also caused excess electrons to combine with molecular oxygen to form ROS during pollen development, thus triggering PCD in the anther; these results are supported by the ROS measurement and TUNEL assay. More recently, research on plant male sterility has increase the focus of attention on ROS metabolism (Li *et al.*, 2012; Luo *et al.*, 2013; K. Wang *et al.*, 2013). The aberrant expression of mitochondrial genes compromised the increased demand for respiratory function and cellular energy in the form of ATP during anther development. The data presented here also show that the same expression patterns are seen in different types of a male-sterile (PHYMS and CMS) wheat line; this provides insights into the molecular mechanisms of male sterility in wheat.

### DEPs are largely implicated in enhanced protein biosynthesis and inhibited degradation of proteins

A total of 20 identified proteins (28% in total) in PHYMS-XN1376 and CMS-XN1376 plants were found to be associated with protein metabolism (Fig. 3B; Supplementary Table S1 at *JXB* online), including molecular chaperones (spots 30–37), protein biosynthesis and degradation (spots 38–43), RNA processing (spots 49 and 50), transcription factors (spots 51 and 52), and nucleotide metabolism (spots 60 and 61), and covered a wide range of DNA replication, RNA transcription, and protein synthesis/processing.

Most mitochondrial proteins are encoded in the nucleus. They are synthesized as precursor forms in the cytosol and must be imported into the mitochondria with the help of different protein translocases. Among these translocases, Tom40, the import channel of the mitochondrial outer membrane, plays an active role in sorting imported proteins. Mitochondrial Hsp70 and chaperonin CPN60 (Hsp60) are mitochondrial chaperones that are typically believed to be responsible for the transportation and refolding of pre-proteins from the cytoplasm into the mitochondrial matrix. Furthermore, the mitochondrial processing peptidase (MPP) removes the pre-sequences from the pre-proteins to form proteins (Becker *et al.*, 2012). Ubiquitin-conjugating enzyme E2 (UBE2) belongs to the ubiquitin-conjugating enzyme family and performs the second step in the ubiquitination reaction that targets a protein for degradation via proteasomes (Miao *et al.*, 2013). In the present study, the co-up-regulation of Tom40 (spots 38 and 39), mtHsp70 (spots 30–33), mtHsp60 (spots 34 and 35), and MPP (spots 40 and 41) was enhanced during novel protein biosynthesis. A variety of proteins (spots 36, 37, 42 49–52, 60, and 61) were directly or indirectly involved in initiation and elongation of the newly growing peptide chains during this synthesis. Conversely, protein degradation was inhibited by the down-regulation of UBE2 (spot 43). Ultimately, this resulted in an increase in the amount and abundance of proteins (Table 1).

### A possible mitochondria-mediated male sterility protein network in wheat

**Fig. 5.**
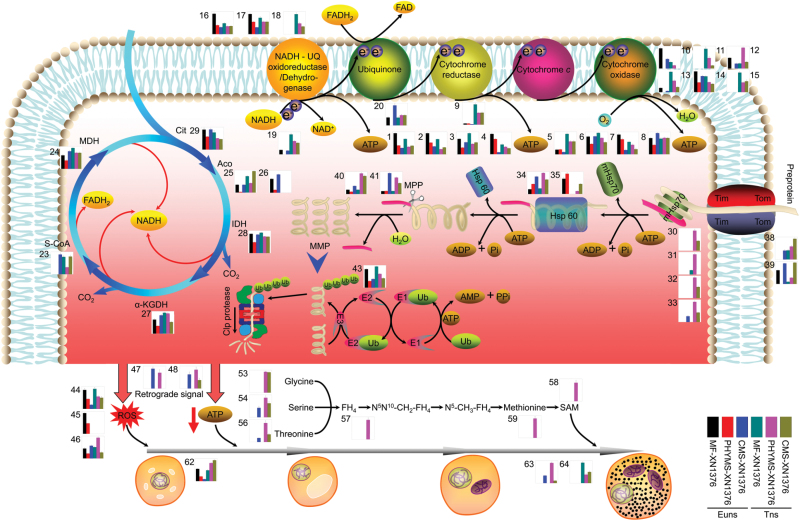
A protein network for the mechanism of mitochondria-mediated male sterility. Histograms and numbers represent protein identification and abundance listed in Supplementary Fig. S5 and Table S1 at *JXB* online. Cit, citrate synthase; Aco, aconitase; IDH, isocitrate dehydrogenase; α-KGDH, α-ketoglutarate dehydrogenase complex; S-CoA, succinyl-CoA synthetase; MDH, malate dehydrogenase; Tom, outer membrane translocases; Tim, import inner membrane translocases; mHsp70, matrix heat shock protein 70; Hsp60, heat shock protein 60; E1, ubiquitin-activating enzymes; E2, ubiquitin-conjugating enzymes; E3, ubiquitin-ligating enzymes; Ub, ubiquitin; FH4, tetrahydrofolate; SAM, *S*-adenosylmethionine; MPP, mitochondrial-processing peptidase; MMP, mature mitochondrial protein; Euns, the early uninucleate stage; Tns, the trinucleate stage.

Plant male sterility plays an important role in developmental and molecular studies and hybrid breeding. In the present study, based on the putative functions and changes in the levels of the 68 identified proteins (Supplementary Fig. S5, S7B; Table S1 at *JXB* online), together with previously published results (Chen *et al.*, 2009, 2010; Zhang *et al.*, 2011, 2013; Ba *et al.*, 2014a, b; Liu *et al.*, 2014), an intriguing mitochondria-mediated male sterility protein network was proposed for wheat (Fig. 5). This network consists of several functional components, including inhibited mtETC and TCA cycle, accelerated biosynthesis and reduced degradation of proteins, generated ROS metabolism, disrupted cell division cycle, and epigenetics.

In the present experiments, mtETC/ATP synthesis-related proteins were preferentially and greatly down-regulated in both PMS-XN1376 and CMS-XN1376 plants from the early uninucleate stage, including one NADH dehydrogenase (spot 19), three complex I proteins (spots 16–18), two complex II proteins (spots 9 and 20), six complex IV proteins (spots 10–15), and eight ATP synthases (spots 1–8). In addition, a range of antioxidant enzymes were down-regulated in this study (spots 44–46; Fig. 4D–F) and in previously published studies (Chen *et al.*, 2009, 2010; Ye *et al.*, 2009). These changes led to excess electrons directly combining with molecular oxygen to generate excessive ROS (Fig. 4A–C) while reducing ATP production in PHYMS-XN1376 and CMS-XN1376 plants. On the one hand, the excessive ROS caused peroxidation of membrane lipids (Fig. 4C) and resulted in increased permeability (Suzuki *et al.*, 2012). In this case, a large number of pre-proteins were rapidly translocated through the outer and inner membranes into mitochondria; there they combined with the up-regulation of Tom40 (spots 38 and 39), mtHsp70 (spot 30–33), mtHsp60 (spots 34 and 35), and MPP (spots 40 and 41), and led to pre-proteins forming proteins; however, the down-regulation of UBE2 (spot 43) inhibited protein degradation. Eventually they increased the amount and abundance of mitochondrial proteins, including elongation factor G (spot 42), RNA helicase (spots 49 and 50), DNA replication licensing factor (spot 60), and site-specific recombinase (spot 61). Moreover, these extra proteins were involved in the biosynthesis of mitochondria-encoded proteins. Thus, the excessive ROS disturbed the balance between protein biosynthesis and degradation, which damaged the equilibrium of protein metabolism in mitochondria, and further affected pollen development. On the other hand, excessive ROS and reduced ATP as the primary signals, combined with the up-regulation of signal transduction proteins (spots 47 and 48), retrograde-regulated pollen development and ultimately led to pollen abortion. Furthermore, three proteins involved in the cell division cycle (spots 62 and 63) and nutrition storage (spot 64) displayed an abnormal change that hindered pollen development (Fig. 1P, T) and the biosynthesis of starch (Fig. 1Q, U); this further aggravated abortion rates. Thus, abnormal cell division and lack of nutrients are also important factors in pollen abortion. The present data also suggest that PHYMS-XN1376 and CMS-XN1376 shared the above pathway with respect to mitochondrial-mediated male sterility.

However, it should be noted that a number of enzymes (spots 23–29), belonging to the TCA cycle, are only down-regulated in PHYMS-XN1376 at the early uninucleate stage and/or at the trinucleate stage. It is possible that the inhibition of TCA cycle enzymes can limit NADH production, energy, and carbohydrate metabolism in PHYMS-XN1376, which indirectly inhibited the mtETC, which has been confirmed by RT-PCR analysis (three energy supply-related genes aconitase, GAPDH, and NFUDP4) and Zhang *et al.* (2011, 2013). This is unlike the abnormal mtETC in CMS-XN1376 plants which was directly inhibited by the related genes. Additionally, a variety of amino acid biosynthesis enzymes (spots 53, 54, and 56) were up-regulated in both PHYMS-XN1376 and CMS-XN1376 plants, and they generated excessive amino acids, including glycine, serine, and threonine. Tetrahydrofolate (spot 57), a one-carbon metabolism carrier only up-regulated in PHYMS-XN1376, converted these excessive amino acids into SAM (spots 58 and 59) which is involved in DNA methylation reactions. This is different from the process in CMS-XN1376 and has been documented by Ba *et al.* (2014b). Therefore, the two abnormal pathways only exist in PHYMS-XN1376 but not in CMS-XN1376.

2-DE analysis and putative functions revealed a highly interconnected protein network, which led to pollen abortion in wheat (Fig. 5). This network was further confirmed by physiological data, RT-PCR, and TUNEL assay (Fig. 4). The results showed that an increase in ROS levels was accompanied by inhibition of antioxidant enzyme activities in both PHYMS-XN1376 and CMS-XN1376 plants from the early uninucleate stage to the trinucleate stage (Fig. 4). Furthermore, RT-PCR analysis indicated that two oxidation stress-related genes (APX and PNDOR) and three energy supply-related genes (aconitase, GAPDH, and NFUDP4) were down-regulated in both PHYMS-XN1376 and CMS-XN1376 (Fig. 4G). These changes imply that the inhibition of mtETC causes excessive ROS generation and a decrease in ATP. Furthermore, the TUNEL assay showed that the PCD in anther tissues was initiated at the early uninucleate stage and extended to the binucleate and trinucleate stages (Fig. 4H; Supplementary Fig. S9 at *JXB* online). These results support the hypothesis that inhibition of mtETC causes excessive ROS generation and a decrease in ATP which induces premature tapetal degeneration and pollen apoptosis, ultimately leading to pollen abortion. Additionally, protein ubiquitination and DNA methylation have been documented by Liu *et al.* (2014) and Ba *et al.* (2014b), respectively.

Taken together, an intimate protein network of male sterility was proposed and confirmed based on proteomic screening and molecular biology. The results provide insight into the molecular mechanism of male sterility in wheat, which gives a useful guide for fertility restoration and the practical application of hybrid breeding.

## Supplementary data

Supplementary data are available at *JXB* online.

Figure S1. Gene expression stability of the seven candidate reference genes for wheat anther using two approaches.

Figure S2. Scanning electron micrograph observations of anthers at the trinucleate stage.

Figure S3. Evaluation of mitochondria fraction.

Figure S4. 2-DE maps analysis of mitochondria proteomes from PHYMS-XN1376, CMS-XN1376, and MF-XN1376.

Figure S5. Abundance profiles of 71 differentially expressed proteins.

Figure S6. Analysis of protein interaction network by the STRING system.

Figure S7. Cellular component (A) and biological pathway (B) networks generated using the BiNGO plugin from the Cytoscape tool.

Figure S8. Analysis of respiratory activity.

Figure S9. Statistical analysis of apoptosis rate (%).

Table S1. Identification of differentially expressed proteins from isolated mitochondria of PHYMS-XN1376, CMS-XN1376, and MF-XN1376.

Table S2. Sixty-eight annotated proteins blasted against the TAIR and ORYSJ protein and TAMTG nucleotide databases.

Table S3. Specific primers used in this study.

Table S4. The abundance of differentially expressed proteins from isolated mitochondria of PHYMS-XN1376, CMS-XN1376, and MF-XN1376.

Table S5. The fold change and *P*-value of differentially expressed proteins in PHYMS-XN1376, and CMS-XN1376.

Table S6. Matched peptide sequences of identified proteins.

Table S7. Corresponding homologues of the three unknown proteins.

Table S8. Abbreviations of the specific protein names in the STRING network (Supplementary Fig. S6).

Table S9. Cellular component of differentially expressed proteins.

Table S10. Biological pathways of differentially expressed proteins.

Supplementary Data
