# Supplementary material for: Comparative studies of mitochondrial proteomics reveal an intimate protein network of male sterility in wheat (Triticum aestivum L.)
Source: J Exp Bot. 2015 Jul 1;66(20):6191–203. doi: 10.1093/jxb/erv322 (PMC4588876; doi:10.1093/jxb/erv322)
Supplement: Supplementary Data [file supp_66_20_6191__index.html]

Comparative studies of mitochondrial proteomics reveal an intimate protein network of male sterility in wheat (Triticum aestivum L.) — Comparative studies of mitochondrial proteomics reveal an intimate protein network of male sterility in wheat (Triticum aestivum L.) — Supplementary Data 

# Comparative studies of mitochondrial proteomics reveal an intimate protein network of male sterility in wheat (*Triticum aestivum* L.)

## Supplementary Data

Data files

- Supplementary Data - Supplementary Data
- Supplementary Data - Supplementary Data
