# Supplementary material for: Comparative studies of mitochondrial proteomics reveal an intimate protein network of male sterility in wheat (Triticum aestivum L.)
Source: J Exp Bot. 2015 Jul 1;66(20):6191–203. doi: 10.1093/jxb/erv322 (PMC4588876; doi:10.1093/jxb/erv322)
Supplement: Supplementary Data [file supp_erv322_jexbot148668_file002.pdf]

Title:

Comparative studies of mitochondrial proteomics reveal an intimate protein network of male sterility in wheat (*Triticum aestivum* L.)

Authors:

Shuping Wang, Gaisheng Zhang, Yingxin Zhang, Qilu Song, Zheng Chen, Junsheng Wang, Jialin Guo, Na Niu, Junwei Wang and Shoucai Ma

Supplementary Fig. S1

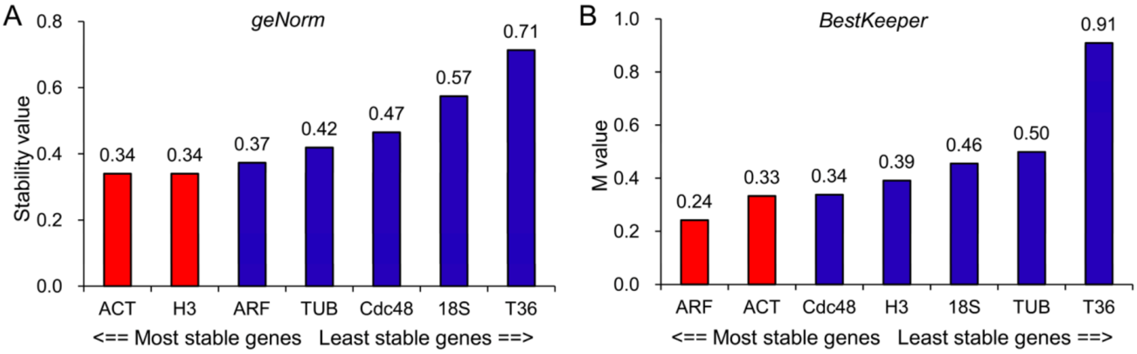

Figure S1. Gene expression stability of the seven candidate reference genes for wheat anther using two approaches. Ranking of individual approaches (BestKeeper and geNorm) as well as consensus ranking are showed. Genes with a high value are less stably expressed compared with genes with a low value. Ribosomal RNA18S (18S), actin (ACT), α-tubulin (TUB), ADP-ribosylation factor (ARF), Cell division cycle 48 (Cdc48), Histone H3 (H3), TaRPII36 (T36). All primers are listed in Supplementary Table S3.

Supplementary Fig. S2

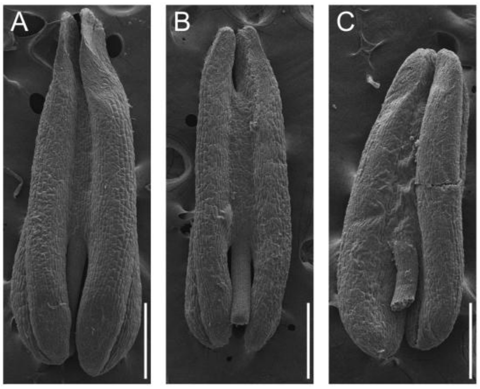

Figure S2. Scanning electron micrograph observations of anthers at the trinucleate stage. (A) MF-XN1376; (B) PHYMS-XN1376; (C) CMS-XN1376. Scale bar: 1 mm.

Supplementary Fig. S3

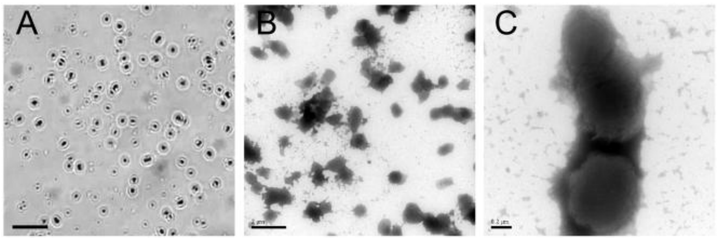

Figure S3. Evaluation of mitochondria fraction. The purified mitochondria were visualized under a light microscope using Janus green B staining (A) and confirmed by electron microscopy (B and C). Scale bar: 10 μm (A), 2 μm (B), 0.2 μm (C).

Supplementary Fig. S4

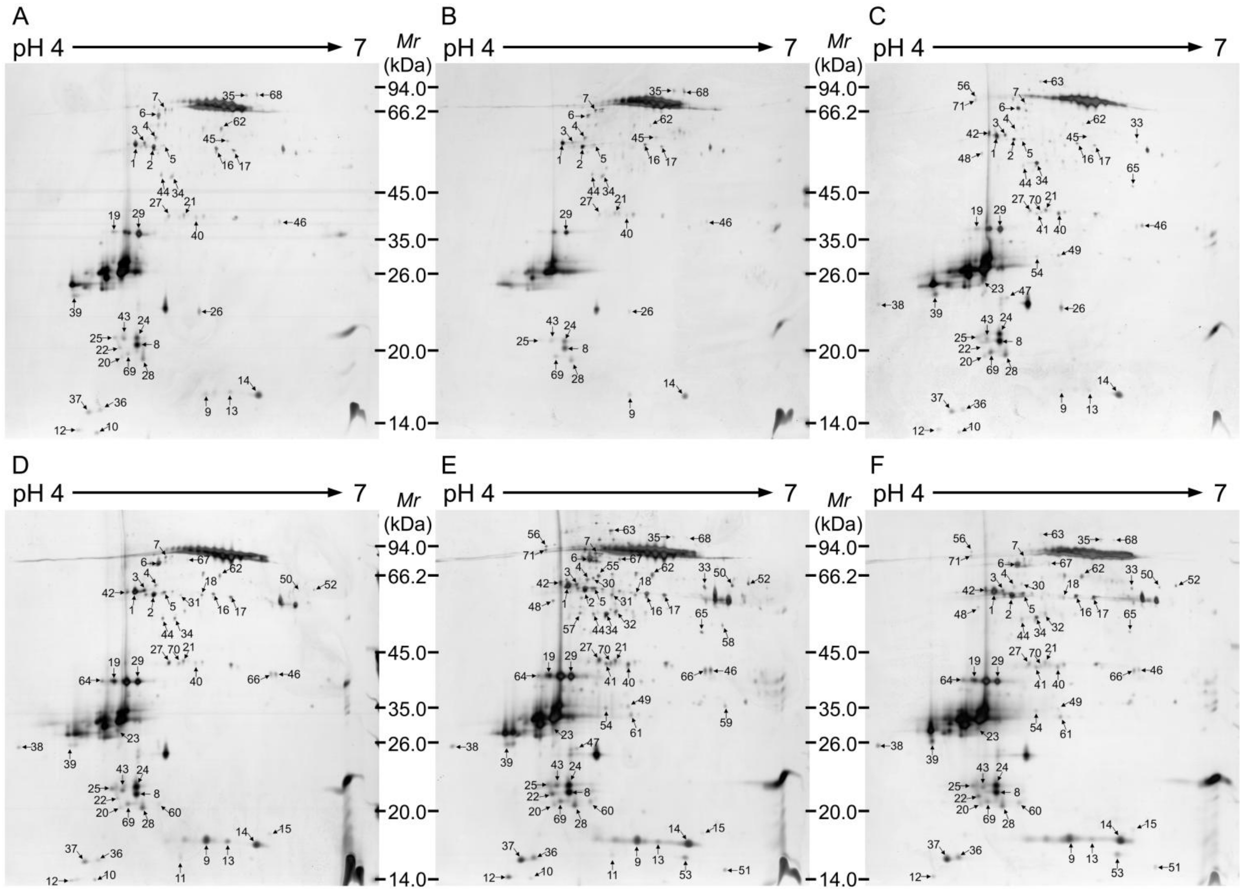

Figure S4. 2-DE maps analysis of mitochondria proteomes from PHYMS-XN1376, CMS-XN1376 and MF-XN1376. (A, B and C) the early-uninucleate stage; (D, E and F) the trinucleate stage. Comparison of protein profiles among MF-XN1376 (A and D), PHYMS-XN1376 (B and E) and CMS-XN1376 (C and F) by two-dimensional electrophoresis (2-DE) gels. Mitochondrial proteins (160 μg) were separated on pH 4–7 linear Immobiline Dry Strip gels (IPG strips) and then on 11% SDS-polyacrylamide gels. Proteins were visualized by silver staining. Numbered spots represent the identifications detailed in Supplementary Table S1.

Supplementary Fig. S5

Figure S5. Abundance profiles of 71 differentially expressed proteins. All data are mean values with SD (n = 3 biological replicates) and listed in Supplementary Table S4.

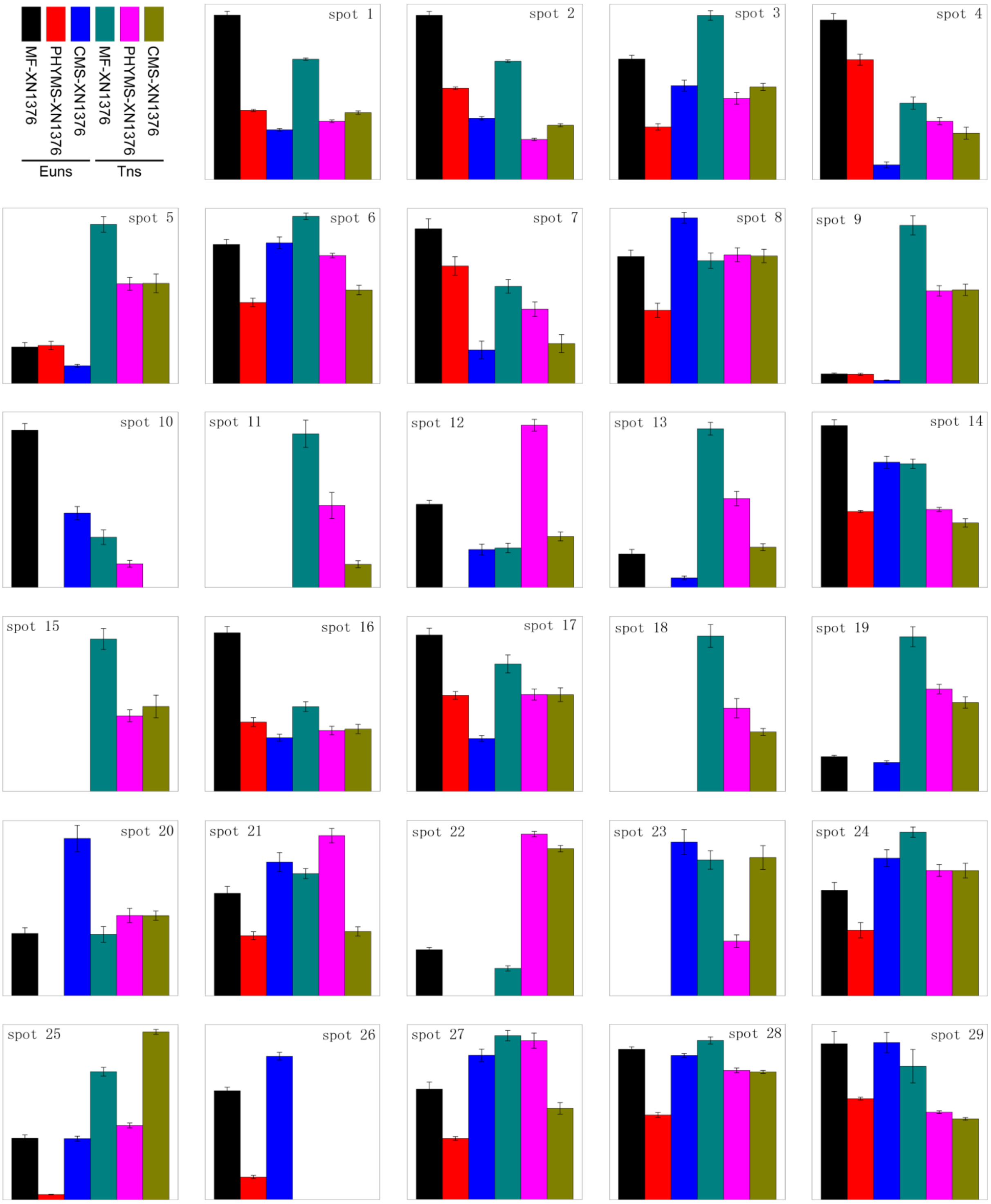

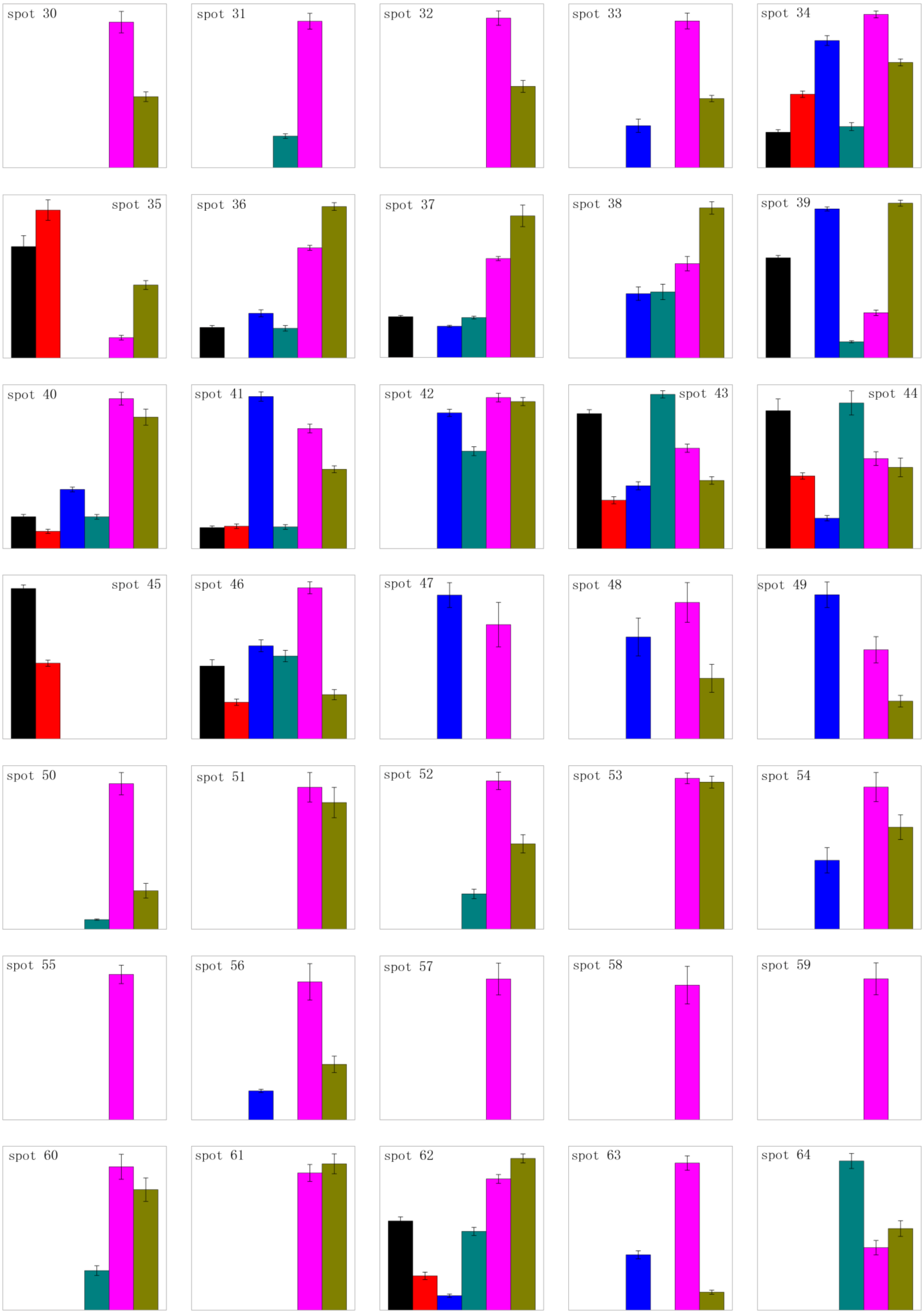
spot 30
spot 31
spot 32
spot 33
spot 34
spot 35
spot 36
spot 37
spot 38
spot 39
spot 40
spot 41
spot 42
spot 43
spot 44
spot 45
spot 46
spot 47
spot 48
spot 49
spot 50
spot 51
spot 52
spot 53
spot 54
spot 55
spot 56
spot 57
spot 58
spot 59
spot 60
spot 61
spot 62
spot 63
spot 64

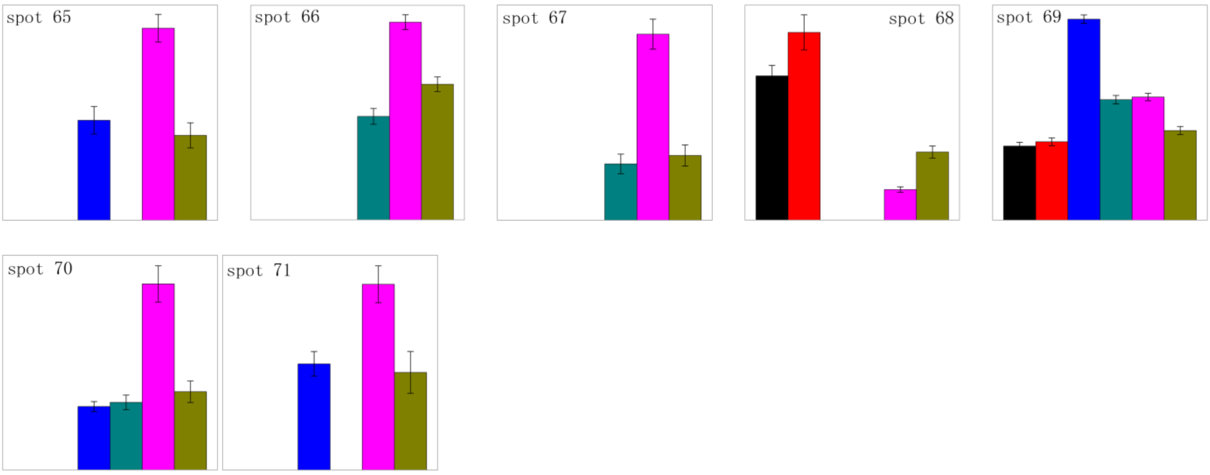

Supplementary Fig. S6

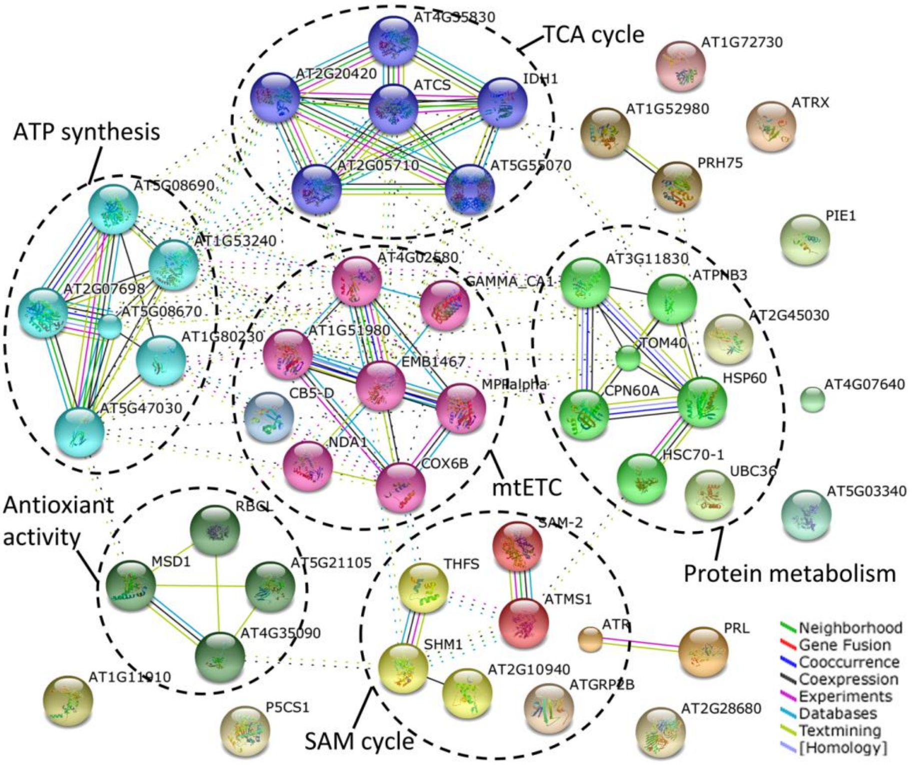

Figure S6. Analysis of protein interaction network by STRING system. TAIR homologous proteins from identified proteins were mapped by searching the STRING 9.1 software with a confidence level of 0.4. Colored lines between the proteins indicate the various types of interaction evidence. Five clusters of highly interacting protein nodes are marked with ellipses and include proteins involved in mitochondrial electron transport (mtETC), ATP synthesis, tricarboxylic acid (TCA) cycle, protein metabolism, S-adenosylmethionine (SAM) cycle, and antioxidant activity.

Supplementary Fig. S7

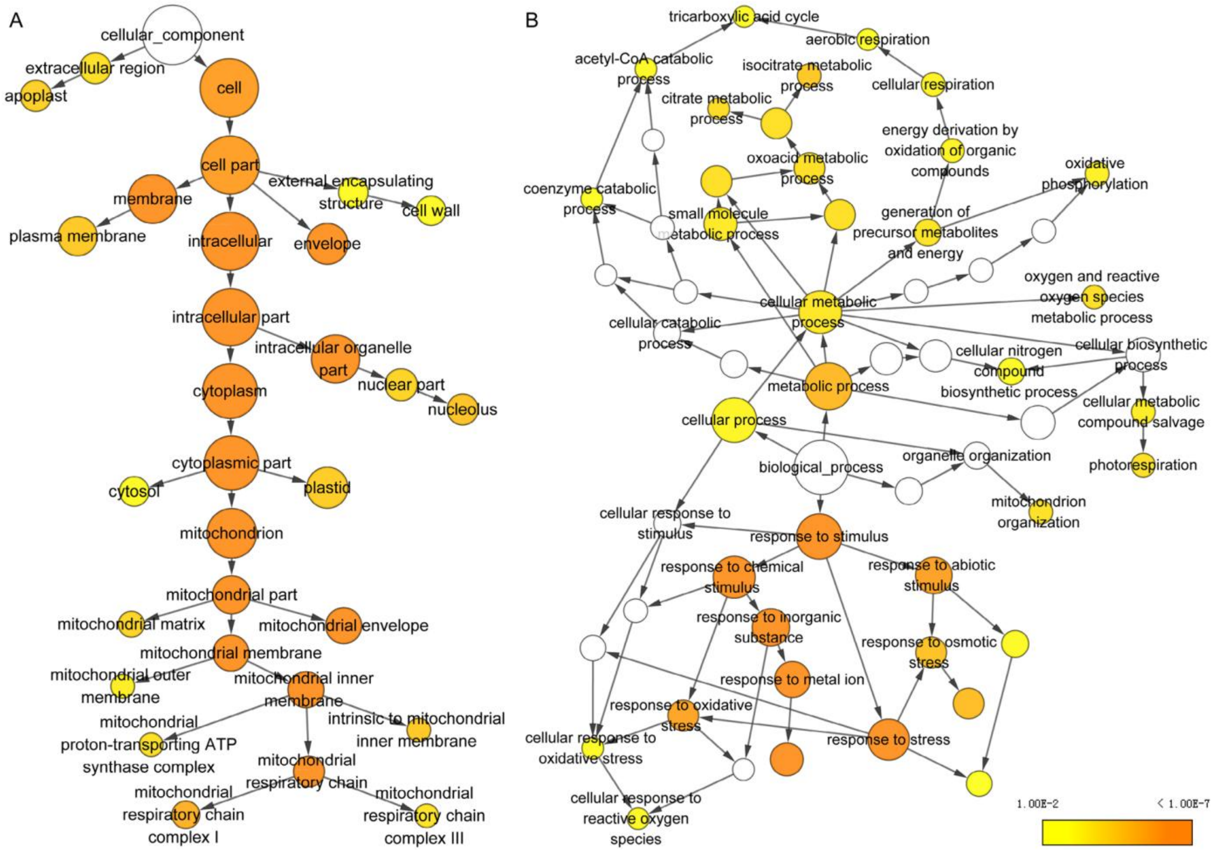

Figure S7. Cellular component (A) and Biological pathway (B) networks generated using BiNGO plugin from Cytoscape tool. Homologous proteins were used for the GO analysis. The node size is proportional to the number of proteins represented by GO category, and color denotes the p-value for each enriched GO term (see the color scale on the right bottom), whereas white nodes are not enriched.

Supplementary Fig. S8

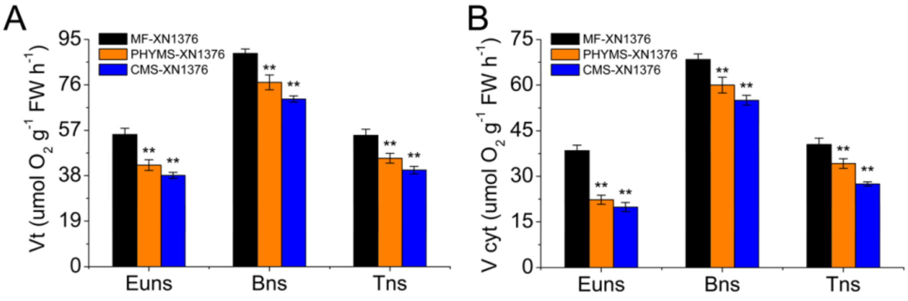

Figure S8. Analysis of respiratory activity. Total respiration (V_t, A) and cytochrome pathway activity (V_{cyt}, B) exhibited a significantly decreased in PHYMS-XN1376 and CMS-XN1376 from the early-uninucleate stage to trinucleate stage. Euns, the early-uninucleate stage; Bns, the binucleate stage; Tns, the trinucleate stage. All data are mean values with SD (*n* = 3 biological replicates). Significant differences (**P* <0.05, ***P* <0.01) between MF-XN1376 and PHYMS-XN1376/CMS-XN1376 were assessed using the Student's *t* test.

Supplementary Fig. S9

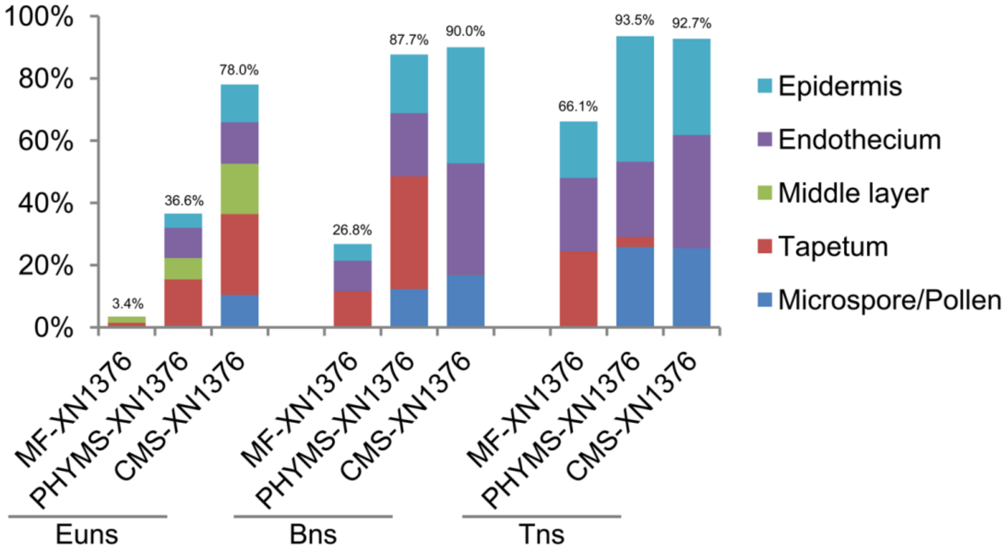

Figure S9. Statistical analysis of apoptosis rate (%). Anthers were stained by the TUNEL assay to determine cell apoptosis and statistical analysis. Data come from Figure 4H and their repeated experiments images (n>20). Euns, the early-uninucleate stage; Bns, the binucleate stage; Tns, the trinucleate stage.
